# Considerations before the application of 5-hydroxymethylation levels of long non-coding RNAs for non-invasive cancer diagnosis

**DOI:** 10.20517/evcna.2021.22

**Published:** 2022-01-21

**Authors:** Zhou Zhang, Chang Zeng, Wei Zhang

**Affiliations:** ^1^Department of Preventive Medicine, Northwestern University Feinberg School of Medicine, Chicago, Illinois 60611, USA.; ^2^The Robert H. Lurie Comprehensive Cancer Center, Northwestern University Feinberg School of Medicine, Chicago, Illinois 60611, USA.

**Keywords:** 5-Hydroxymethylcytosine, long non-coding RNA, cell-free DNA, cancer biomarker

## Abstract

Previous studies have suggested that aberrant 5-hydroxymethylcytosines (5hmC) modifications are related to cancer pathobiology. Genome-wide profiling 5hmC in circulating cell-free DNA (cfDNA) using the highly sensitive chemical labeling-based 5hmC-Seal technique has been demonstrated to have the potential to be a robust epigenomic tool for cancer biomarker discovery. Prior studies have mostly focused on cfDNA-derived 5hmC-Seal data summarized in well-annotated genic features (e.g., gene bodies) or unbiased bins. Zhou *et al*. recently proposed long non-coding RNAs (lncRNAs) as an alternative molecular target for biomarker discovery using publicly available 5hmC-Seal data. Considering its potential clinical impact, we would like to comment on Zhou *et al*. and advocate more serious consideration of critical issues such as the availability of clinical information and technical variables, especially when performing secondary analysis using publicly available data, with the aim of improving data transparency and translatability.

The 5-hydroxymethylcytosine (5hmC) is an emerging epigenetic marker that reflects gene activation status^[1]^. Previous studies have suggested that aberrant 5hmC modifications are related to cancer pathobiology. Genome-wide profiling of 5hmC in circulating cell-free DNA (cfDNA) using the 5hmC-Seal technique^[2]^, a highly sensitive chemical labeling approach suitable for a very limited amount of clinical biospecimens (e.g., 1-2 ng of cfDNA from a few mL of plasma) has been demonstrated by our team and other groups to be a robust epigenomic tool for cancer biomarker discovery with the goal of achieving non-invasive cancer diagnosis and prognosis^[3-6]^.

Analytically, although our previous studies mostly focused on the 5hmC-Seal profiles summarized in well-annotated genic features (e.g., gene bodies) or unbiased bins, recently, we started exploring the possibility of integrating 5hmC profiles summarized for long non-coding RNAs (lncRNAs) and repetitive elements to improve biomarker discovery using glioblastoma (GBM) as an example^[3]^. Specifically, in the cell, lncRNAs are known to regulate gene expressions at both transcriptional and post-transcriptional levels, and play important and heterogeneous regulatory roles in nearly all cellular and biological processes, including transcriptions, translation, and nuclear trafficking, as well as tumorigenesis and therapy resistance^[7]^. In GBM, dysregulation of lncRNAs can contribute to the epithelial-mesenchymal transition, therefore promoting cancer metastasis^[8]^. In addition, a recent study reported a positive association between 5hmC and lncRNA transcription in colorectal cancer, indicating the regulatory role of 5hmC on lncRNA expression^[9]^. Given its tissue-specificity and roles in tumor initiation, progression and resistance to therapy, lncRNAs remain to be promising markers for cancer diagnosis and prognosis.

Specifically, we read with interest that a recent study published by Zhou *et al.*^[10]^ described the development of plasma-derived 5hmC-LncRNA diagnostic score (5hLD-score) for cancer diagnosis and surveillance using publicly available 5hmC data. The proposed 5hLD-score was shown the capability of distinguishing tumors from healthy controls in their training and internal validation cohorts. Further validation showed the 5hLD-score achieved area under the curve (AUC) of 0.85, 0.89, and 0.77 in a non-small cell lung cancer cohort, an esophageal cancer cohort, and a hepatocellular carcinoma (HCC) cohort, respectively. The authors identified an association between the 5hLD-score and the progression of liver cancer in the HCC cohort, as well as the capability to identify the origin and location of tumors. This study further supported the clinical potential of 5hmC levels in lncRNAs for cancer early detection and progression monitoring. However, we would like to comment on a few important issues of Zhou *et al*. and advocate that there are several critical issues that need to be taken into consideration in order to make an informed conclusion of the current status of applying 5hmC levels in lncRNAs as a marker for cancer diagnosis and prognosis, especially when such a conclusion was drawn from performing secondary data analysis using public data.

Firstly, during statistical modeling, differential 5hmC modifications should be identified in the training set solely. Instead, Zhou *et al*. used the whole Li’s cohort (training and internal validation set combined) to perform the differential analysis. This procedure would have caused data leakage, which introduced the knowledge of the validation set into the modeling process, and could have led to model overfitting in the validation set. Therefore, the observed differences in terms of the AUCs between the internal validation set and the independent validation set presented by Zhou *et al*. could be due to data leakage and model overfitting, which should be evaluated using appropriate tests such as the Delong test^[11]^.

Secondly, when using the 5hmC profiles generated from different platforms/protocols, sequencing length, depth, or platform information should be taken into considerations. Regarding these potential technical biases, Zhou *et al*. did not take them into considerations in their analysis. To our best knowledge, the Li’s cohort^[6]^ was sequenced with 150 base-pair (bp) paired-end library, while the Cai’s liver cancer cohort^[5]^ was sequenced with 38 bp paired-end library. In addition, the publicly available 5hmC data were generated at different times and core facilities. Those unaccounted factors, taken together, could cause substantial batch effects, with the likelihood of leading to misinterpretation of the results.

Thirdly, clinical variables, such as age, gender, tumor stages, place of residence, and lifestyle, have been established as potential confounders in epigenetic studies. These variables (known or hidden) contribute to the epigenetic differences between cases and controls. Not appropriately adjusting for these confounding variables could lead to biased interpretation of results. For examples, in figure 5, Zhou *et al*.^[10]^ argued that the 5hLD-scores were associated with liver cancer progression. However, this finding could be confounded by patient’s age, as the liver cancer patients were much older than patients with hepatitis B infection history in the Cai cohort^[4]^.

Finally, unlike mRNAs with protein-coding potential or microRNAs with high sequence conservations, lncRNAs possessing unique features such as lower transcription rate, reduced stability and lower expression levels can pose analytic constraints in the characterization and annotation of lncRNAs^[12]^. For example, the GENCODE^[13]^ lncRNAs were identified from RNA-Seq data and algorithm not optimized for the full exploitation and annotation for non-polyA lncRNA transcripts or functional lncRNAs with relatively lower expression. Furthermore, given the relatively lower expression of lncRNA in non-brain tissue types, the signal to noise ratios of 5hmC mapping over lncRNA regions on cfDNA are expected to be even lower in non-brain cancer patients included at least in theory. However, Zhou *et al*. did not provide any evaluation of the expression levels or tissue-specificity of these lncRNAs before proceeding to the marker discovery phase. As a result, the 5hmC profiles of lncRNAs in the current study could have been subjected to random noise due to low abundance. Last but not least, we observed synergistic effects between the 5hmC of lncRNAs and other genomic feature types (i.e., gene body, repetitive elements and histone marks) in our GBM study^[3]^, it would be interesting if future studies could incorporate other genomic feature types and compare the performance by feature type, separately and integratively.

In conclusion, in our opinion, the 5hmC levels of lncRNAs could be a promising biomarker for cancer diagnosis and monitoring, though future large studies of individuals with more comprehensive clinical, pathological, and epidemiological information, as well as the application of more robust data analysis plans (e.g., consideration of hidden variables) will help improve data transparency and provide more insights into the translatability of these molecular targets.

## DECLARATIONS

### Copyright

© The Author(s) 2022.
